# Recombination in feline immunodeficiency virus from feral and companion domestic cats

**DOI:** 10.1186/1743-422X-5-76

**Published:** 2008-06-17

**Authors:** Jessica J Hayward, Allen G Rodrigo

**Affiliations:** 1 Bioinformatics Institute, Allan Wilson Centre for Molecular Ecology and Evolution, School of Biological Sciences, The University of Auckland, Auckland, New Zealand

## Abstract

**Background:**

Recombination is a relatively common phenomenon in retroviruses. We investigated recombination in *Feline Immunodeficiency Virus *from naturally-infected New Zealand domestic cats (*Felis catus*) by sequencing regions of the *gag*, *pol *and *env *genes.

**Results:**

The occurrence of intragenic recombination was highest in *env*, with evidence of recombination in 6.4% (n = 156) of all cats. A further recombinant was identified in each of the *gag *(n = 48) and *pol *(n = 91) genes. Comparisons of phylogenetic trees across genes identified cases of incongruence, indicating intergenic recombination. Three (7.7%, n = 39) of these incongruencies were found to be significantly different using the Shimodaira-Hasegawa test.

Surprisingly, our phylogenies from the *gag *and *pol *genes showed that no New Zealand sequences group with reference subtype C sequences within intrasubtype pairwise distances. Indeed, we find one and two distinct unknown subtype groups in *gag *and *pol*, respectively. These observations cause us to speculate that these New Zealand FIV strains have undergone several recombination events between subtype A parent strains and undefined unknown subtype strains, similar to the evolutionary history hypothesised for HIV-1 "subtype E".

Endpoint dilution sequencing was used to confirm the consensus sequences of the putative recombinants and unknown subtype groups, providing evidence for the authenticity of these sequences. Endpoint dilution sequencing also resulted in the identification of a dual infection event in the *env *gene. In addition, an intrahost recombination event between variants of the same subtype in the *pol *gene was established. This is the first known example of naturally-occurring recombination in a cat with infection of the parent strains.

**Conclusion:**

Evidence of intragenic recombination in the *gag*, *pol *and *env *regions, and complex intergenic recombination, of FIV from naturally-infected domestic cats in New Zealand was found. Strains of unknown subtype were identified in all three gene regions. These results have implications for the use of the current FIV vaccine in New Zealand.

## Background

*Feline Immunodeficiency Virus *(FIV) is a retrovirus that specifically infects felines and hyaenas [1]. In the strain that infects the domestic cat, *Felis catus *(FIV-Fca), five phylogenetic subtypes have been described, labelled A to E [2-4]. These subtypes are based on the V3 to V5 region of the *env *gene, with some recent studies confirming phylogenies using the *gag *gene [2,5-8]. In the *env *gene, intrasubtype pairwise distances of up to about 14% are observed, while intersubtype distances of 15% to 38% are commonly found [2,4,8]. Recently, we and others have proposed potentially novel subtypes based on phylogenetically-distinct sequence groups that differ from known subtypes [5,9,10].

The *env *gene encodes the viral surface and transmembrane glycoproteins, gp120 and gp41 respectively. These glycoproteins are involved in host cell recognition and have been shown to contain epitopes that elicit cell-mediated and humoral immune responses in FIV-infected individuals [11,12]. As this region of the viral genome is likely to be under strong selection, it is likely to change rapidly.

As a retrovirus, FIV has a relatively high evolutionary rate, on the order of 1–3% per decade in the *env *and *pol *genes of the cougar strain, FIV-Pco [13]. This high rate is largely attributed to substitution errors made during reverse transcription [14]. However, as a retrovirus with a diploid genome, the phenomenon of recombination is also very important in the evolution of FIV. Recombination occurs as a result of template-switching by the enzyme *reverse transcriptase *when the particular infecting virion has a heterozygous genome [15]. Hence, a prerequisite for recombination is dual infection. Previously, three naturally-infected cats have been identified with FIV intersubtype dual infections: one with subtypes A and B from USA [16], and two with subtypes A and C from New Zealand (NZ) [17]. In addition, a natural superinfection event has been documented between two subtype A FIV-infected cats housed together in Australia [18]. The superinfected cat has its own unique FIV strains that had been isolated from the cat previously and additional FIV strains that show within 6.8% nucleotide similarity to the FIV strains isolated from the cat inhabiting the same house [18]. Naturally-occurring FIV-Fca intersubtype recombination has been described in domestic cats from Canada, Hawaii, USA and Japan [6,16,19].

In our previous study of FIV *env *gene sequences in NZ domestic cats, we found subtypes A and C, nine putative A/C recombinants and a group of sequences of an unknown and phylogenetically-distinct subtype [10]. Here we further investigate the occurrence of recombinant sequences in naturally-infected NZ domestic cats using the *gag *and *pol *genes, in addition to *env*. We find an additional intragenic recombinant in the *env *gene, giving ten recombinant sequences for this gene, or 6.4% (n = 156). From each of the *gag *and *pol *gene regions we find only one (2.1% and 1.1% respectively) intragenic recombinant. Our results also show that the previously-proposed unknown subtype sequences from the *env *gene do not show the same monophyletic grouping in the *gag *and *pol *regions. In addition, we find inconsistencies in subtype C designation in the *gag *and *pol *genes. We suggest that the patterns seen in these samples are the result of multiple recombination events throughout the FIV genome. The results illustrate the importance of sequencing and analysing multiple genes to gain a more complete picture of recombination events.

## Results

### Phylogenetic trees

**Figure 1:**
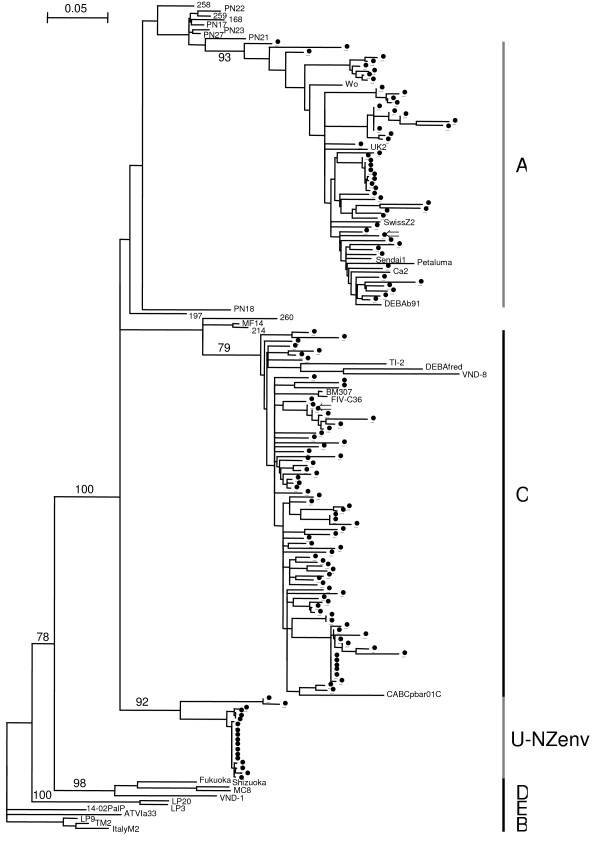
**NJ phylogenetic tree of *env *sequences from NZ domestic cats**. Tree is rooted by subtype B. Subtypes are shown along the right side of the tree. Bootstrap values based on 1000 replicates are shown for the major groups. Sequences with ⇐ are used as reference sequences in the RIP analyses. Outlier sequences are 258, PN22, 259, 168, PN17, PN23, PN27, PN21, PN18, 197, 260, MF14, 214. U-NZenv is a group of NZ sequences that does not group with a known subtype. Reference sequences from GENBANK are; subtype A: Sendai1 (D37813, Japan), Petaluma (M25381, USA), DEBAb91 (AF531043, Germany), UK2 (X69494, UK), SwissZ2 (X57001, Switzerland), Wo (L06135, France) and Ca2 (DQ873714, South Africa); subtype B: TM2 (M59418, Japan), ItalyM2 (X69501, Italy), ATVIa33 (AF531045, Austria), LP9 (D84497, Argentina) and 14-02PalP (DQ072558, Portugal); subtype C: CABCpbar01C (U02393, Canada), TI-2 (AB016026, Taiwan), DEBAfred (U57020, Germany), BM3070 (AF474246, Canada), FIV-C36 (AY600517, USA) and VND-8 (AB083509, Vietnam); subtype D: MC8 (D67062, Japan), Shizuoka (D37811, Japan), Fukuoka (D37815, Japan) and VND-1 (AB083502, Vietnam); subtype E: LP3 (D84496, Argentina) and LP20 (D84498, Argentina).

The *env *NJ phylogenetic tree (Fig. 1) shows three main groups, which are subtypes A, C and an unknown (U-NZenv), as previously shown [10]. One further sequence, in addition to the 17 sequences from our earlier study, now falls in the unknown group. Average K2P distances suggest that the unknown group could be a new subtype, with distances ranging from 20.6% to 22.7% between the unknown and other subtypes (Table 1). Also seen in the *env *NJ tree are 13 outliers, 11 of which were documented in our previous study [10].

**Table 1:** Average percentage K2P nucleotide distances between and within all *env *sequences of different subtypes included in this study, as shown in Fig. 1

	Subtype A	Subtype B	Subtype C	Subtype D	Subtype E	U-NZenv
Subtype A	8.07					
Subtype B	23.04	9.82				
Subtype C	23.54	21.36	7.38			
Subtype D	23.03	17.94	22.08	8.91		
Subtype E	23.31	15.84	23.22	19.03	5.03	
U-NZenv	21.44	21.69	20.63	22.73	22.06	2.44

**Figure 2:**
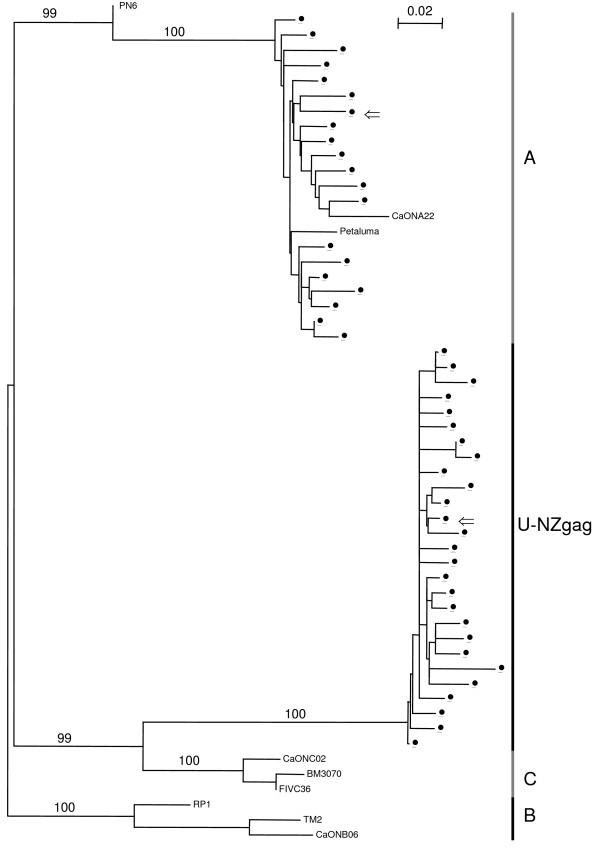
**NJ phylogenetic tree of *gag *sequences from NZ domestic cats**. Tree is rooted by subtype B. Subtypes are shown along the right side of the tree. Bootstrap values based on 1000 replicates are shown for the major groups. Sequences with ⇐ are used as reference sequences in the RIP analyses. Outlier sequence is PN6. U-NZgag is a group of NZ sequences that does not group with a known subtype. Reference sequences from GENBANK are; subtype A: CaONA22 (AY369383, Canada), Petaluma (M25381, USA); subtype B: CaONB06 (AY369381, Canada), TM2 (M59418, Japan), RP1 (AJ304962, Portugal); subtype C: BM3070 (AF474246, Canada), FIVC36 (AY600517, USA), CaONC02 (AY369384, Canada).

The *gag *NJ tree (Fig. 2) features two large groups of NZ sequences, one of which is subtype A. The other group of sequences (U-NZgag) is monophyletic with subtype C reference sequences (BM3070 and CaONC02 from Canada, and FIV-C36 from USA). However, U-NZgag sequences are quite distantly related to the subtype C sequences, with an average K2P distance of 15.64%, a value greater than intrasubtype *gag *distances and consistent with intersubtype *gag *distances (Table 2). This level of similarity indicates that the U-NZgag and C groups may be distinct but related subtypes.

**Table 2:** Average percentage K2P nucleotide distances between and within all *gag *sequences of different subtypes included in this study, as shown in Fig. 2

	Subtype A	Subtype B	Subtype C	U-NZgag
Subtype A	3.58			
Subtype B	15.03	6.32		
Subtype C	13.38	16.95	2.49	
U-NZgag	17.33	16.12	15.64	2.66

Interestingly, when we include the four subtype C isolates from Taiwan (TI-1 (AB027298), TI-2 (AB027299), TI-3 (AB027300), TI-4 (AB027301)) [20] by cropping about 550 bp from the start of our *gag *sequence alignment, we find that the Taiwan isolates group with the U-NZgag sequences (average K2P distance of 3.74%; tree not shown). This finding adds weight to the validity of the U-NZgag sequences and also suggests a common origin between NZ and Taiwanese FIV.

**Figure 3:**
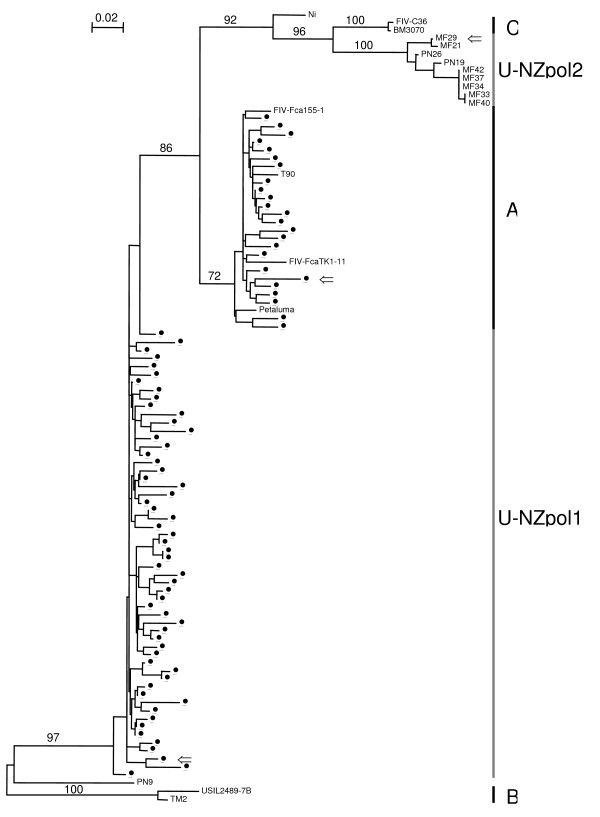
**NJ phylogenetic tree of *pol *sequences from NZ domestic cats**. Tree is rooted by subtype B. Subtypes are shown along the right side of the tree. Bootstrap values based on 1000 replicates are shown for the major groups. Sequences with ⇐ are used as reference sequences in the RIP analyses. Outlier sequences are Ni and PN9. U-NZpol1 and U-NZpol2 are groups of NZ sequences that do not group with a known subtype. Reference sequences from GENBANK are; subtype A: T90 (S67753, Australia), Petaluma (M25381, USA), FIV-Fca155-1 (U53760, Argentina), FIV-FcaTK1-11 (U53762, England); subtype B: TM2 (E03581, Japan), USIL2489 (U11820, USA); subtype C: BM3070 (AF474246, Canada), FIV-C36 (AY600517, USA).

The *pol *NJ tree (Fig. 3) shows NZ sequences split into three groups. One of these is subtype A. The other two groups of sequences (U-NZpol1 and U-NZpol2) do not belong to a known subtype. Although U-NZpol2 forms a monophyletic group with subtype C, the average K2P distance of 8.88% (Table 3) is higher than all other *pol *intrasubtype distances. RIP analyses also show that the nine sequences belonging to U-NZpol2 are most similar to subtype C, with strongest similarity in the second "half" of the sequences (data not shown).

**Table 3:** Average percentage K2P nucleotide distances between and within all *pol *sequences of different subtypes included in this study, as shown in Fig. 3

	Subtype A	Subtype B	Subtype C	U-NZpol1	U-NZpol2
Subtype A	3.69				
Subtype B	14.57	2.99			
Subtype C	12.82	15.63	0.23		
U-NZpol1	8.33	14.04	13.32	2.66	
U-NZpol2	13.41	17.43	8.88	14.20	2.37

The largest group of NZ *pol *sequences, U-NZpol1, is most closely related to subtype A (Table 3). This cluster of sequences does not form a monophyletic group. However, when the recombinant sequence PN9 is removed and a NJ tree reconstructed, U-NZpol1 does form a monophyletic group (data not shown). RIP analysis shows that this group of sequences look like recombinants of subtype A and C. However, KH test results are not significant for these sequences (data not shown).

### Intragenic recombination

#### Env

**Figure 4:**
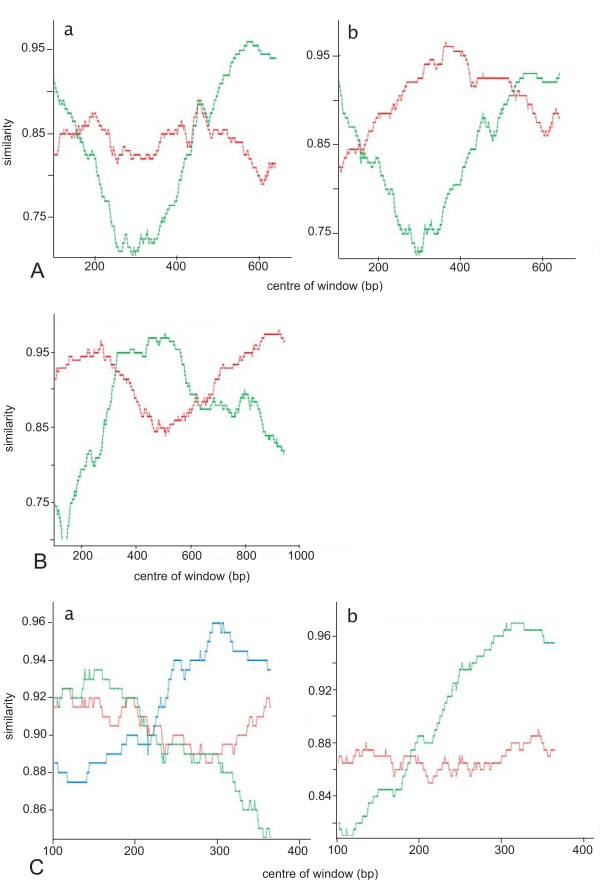
**RIP outputs**. Reference sequences used are highlighted in the respective NJ trees in Figs. 1, 2, 3. (A) Two new outlier *env *sequences. (a) PN18; (b) PN27. Red is similarity to subtype A, green is similarity to subtype C. (B) *gag *outlier sequence, PN6. Red is similarity to subtype A, green is similarity to U-NZgag. (C) *pol *outlier sequences. (a) Ni: red is similarity to subtype A, green is similarity to U-NZpol1, blue is similarity to U-NZpol2. (b) PN9: red is similarity to subtype B, green is similarity to U-NZpol1.

RIP outputs show that the two new *env *outliers are potential recombinant sequences, with clear crossover patterns (Fig. 4A). The Kishino-Hasegawa (KH) test shows significant results for both halves of the query sequence for one of these additional outliers (PN18; data not shown).

From the RIP alignments and visual inspection of the sequences, the approximate locations of the putative crossover points for each *env *putative recombinant sequence were determined. Of the ten putative recombinant sequences (nine from our previous study and one from the present study), there are only six unique recombinant patterns, all involving subtypes A and C (Table 4). Four cats share one recombinant pattern with two crossovers and two cats share another with only a single crossover. One particular crossover site (range of 7508–7522) is featured in six NZ sequences.

**Table 4:** Crossover patterns in the ten *env *putative recombinant sequences

Recombination subtypes	Crossover one (bp)^	Crossover two (bp)^	NZ Sample
C/A/C	7518–7520	7975–8012	168 259 PN17 PN23
C/A/C	7508–7522	7831–7847	258
C/A/C	7518–7520	7796–7799	PN18
A/C	7616–7617		214 MF14
A/C	7723–7739		197
A/C	7882–7884		PN21

^ relative to Petaluma

From a total of 156 FIV-infected NZ cat *env *sequences subjected to phylogenetic analysis, thirteen outliers were identified. Ten of the thirteen outlier sequences had significantly different topologies constructed from each side of the crossover site as determined by the KH test. Thus, ten (6.4%) putative recombinants were found.

**Figure 5:**
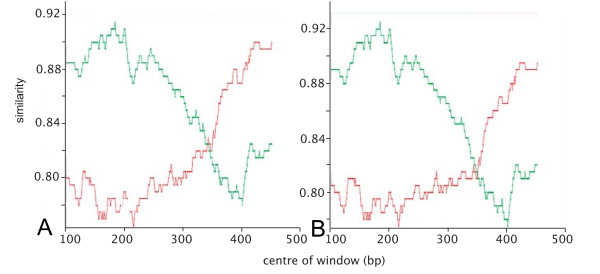
**RIP outputs for *env *sequences from Duarte & Tavares (2006)**. (A) 150_02LisP; (B) 164_02UZP. Red is similarity to reference subtype A (Petaluma); green is similarity to reference subtype B (TM2).

Given the level of recombination that we have found in *env*, we suspect that there are a significant number of undetected putative recombinant sequences in public databases, incorrectly identified as belonging to a particular subtype. For example, a recent study on FIV strains in cats of Portugal found two outlying *env *sequences (150_02LisP and 164_02UZP) that were initially assigned into subtype A [9]. However, genetic divergence analysis and an amino acid phylogenetic tree indicated that the correct location was in a subcluster of subtype B [9]. We analysed the two suspicious sequences using RIP and the KH test, and found that they both show a statistically significant intragenic B/A recombination event (Fig. 5; Table 5).

**Table 5:** KH test results for *env *sequences 150_02LisP and 164_02UZP from Duarte & Tavares (2006)

	First "half"		Second "half"	
Sample	Diff -lnL	P-value	Diff -lnL	P-value

150_02LisP	71.850	0.000*	56.901	0.000*
164_02UZP	73.500	0.000*	52.138	0.000*

* indicates significance at the 0.05 level

#### Gag

There is only a single outlier sequence in the *gag *NJ tree (PN6; Fig. 2). This was tested for recombination using RIP and shows a clear recombination pattern, with two crossover events (Fig. 4B). The KH test results confirm that this sequence is a putative recombinant (Table 6). Ten endpoint dilution sequences were obtained for the *gag *region of PN6, all of which group with the consensus sequence on a NJ phylogenetic tree with a maximum pairwise distance of 1.42%. From a total of 48 FIV-infected NZ cat *gag *sequences subjected to phylogenetic analysis, one (2.1%) putative recombinant was found.

**Table 6:** KH test results for *gag *sequence (PN6) and *pol *sequences (Ni, PN9)

	First "half"		Second "half"	
		
Sample	Diff -lnL	P-value	Diff -lnL	P-value
PN6	35.337	0.002*	26.019	0.004*
Ni	3.935	0.447	32.334	0.000*
PN9	19.552	0.002*	45.098	0.000*

*indicates significance at the 0.05 level

#### Pol

There are two outlier sequences on the *pol *NJ tree (Ni, PN9; Fig. 3). The RIP output for Ni shows a recombination event between A/U-NZpol1 and U-NZpol2 (Fig. 4C(a)), although this is not statistically significant (Table 6). PN9, in contrast, suggests a recombination event between subtype B and U-NZpol1 (Fig. 4C(b)), which is statistically significant (Table 6). Note that although PN9 shows statistical significance between the two sides of the putative crossover location, the similarity values to subtype B in the first part of the sequence are low. Only one (1.1%) putative recombinant *pol *sequence is found from analysing 91 samples in this region. This sequence is the only recombinant involving subtype B found in a NZ cat.

### Intergenic recombination

**Figure 6:**
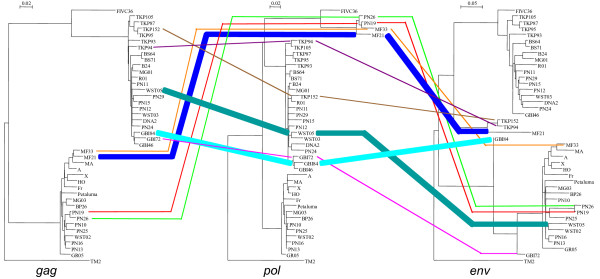
***Gag, pol *and *env *ML trees of sequences for which all three genes were amplified**. Lines between the trees link sequences from the same sample, to highlight incongruencies. The three thickened lines are the statistically significant intergenic recombinant sequences. Reference sequences used are: subtype A (Petaluma, M25381); subtype B (TM2, M59418); subtype C (FIV-C36, AY600517).

The incongruent subtype assignment seen in different gene regions of nine NZ cat samples are shown in Fig. 6. The ML tree that was determined to best describe the data and thus used as the backbone constraint in further SH tests, is the concatenated gene tree.

Significant results from the SH tests to determine differences in ML trees constructed with the inclusion of putative intergenic recombinant sequences are shown in Table 7. After the Bonferroni correction was applied, only five pairs of ML trees returned statistically significant results for the reciprocal SH tests. None of the significant results involve the *pol *region.

**Table 7:** Summary of the statistically significant SH test results, at the 0.05 level

Sample	Gene region	Subtype	Diff. -log likelihood	P-value	Bonferroni correction
MF21	*gag*	A	30.56	0.004	0.016
	*env*	U-NZenv	40.14	0.001	0.004
GBI84	concatenated	Outlier	82.23	0.000	0.000
	*gag*	U-NZgag	35.70	0.003	0.012
GBI84	*gag*	U-NZgag	35.70	0.003	0.012
	*env*	A	150.36	0.000	0.000
WST05	concatenated	Outlier	175.59	0.000	0.000
	*gag*	U-NZgag	25.68	0.012	0.048
WST05	*gag*	U-NZgag	25.67	0.012	0.048
	*env*	A	250.69	0.000	0.000

Three of the five statistically significant SH test results were between the gene trees for *gag *and *env*. Therefore, in these three samples we have evidence of putative intergenic recombination.

Two of these samples (GBI84, WST05) also gave statistically significant results for incongruencies between the concatenated tree and the *gag *gene tree. As a conservative estimate, at least three of the 39 samples, or 7.7%, are intergenic recombinants.

### Unknown subtype

Representatives of the samples that make up the unknown subtype of the *env *NJ tree were sequenced in the *gag *and *pol *gene regions in order to confirm the existence of a novel NZ-specific subtype. However, in the *gag *NJ tree, the U-NZenv samples are found in subtype A and the U-NZgag group. In the *pol *NJ tree, the U-NZenv sequences are found in U-NZpol1 and U-NZpol2 groups. Thus, we have no consistent evidence for a novel NZ-specific subtype across the three gene regions. Note that, due to low viral load and sample quantity limitations, not all the sequences of *env *unknown subtype have been amplified in the *gag *and *pol *genes.

The discrepancies in the placement of the sequences of unknown subtype seen between the phylogenetic trees could be explained by multiple recombination events between subtype A and undefined parent strains. This possible explanation is similar to that suggested for HIV-1 subtype E (CRF_AE01), which is hypothesised to be a circulating recombinant form of subtype A and an unknown parent strain (subtype E) that has not been found [21].

### Endpoint dilution sequencing

With the exception of two samples (see next section), all endpoint dilution sequences and the respective consensus sequence of the same sample are within 2.35% of each other (Table 8). This shows that each consensus sequence is a representative of a true proviral sequence and not an example of PCR-mediated recombination as a result of dual infection.

**Table 8:** Results of *env *and *pol *endpoint dilution sequencing, showing the maximum pairwise distance sequences from the same sample, including the consensus sequence (using GTR with rates = Γ and shape = 0.5)

Sample	*Env *subtype*	No. *env *endpoint sequences^	Maximum pairwise distance (%)	*Pol *subtype*	No. *pol *endpoint sequences^	Maximum pairwise distance (%)
168	PR	12#	0.510	A	nd	
258	PR	11#	0.524	U-NZpol1	nd	
259	PR	10#	0.873	U-NZpol1	nd	
260	PR	11	0.249	U-NZpol1	nd	
GBI46	C	nd		U-NZpol1	10	0.442
GBI72	A	nd		U-NZpol1	10	0.660
GBI84	A	nd		U-NZpol1	10	0.667
MF21	U-NZenv	nd		U-NZpol2	10	0.661
MF29	U-NZenv	nd		U-NZpol2	13	1.844
PN9	C	nd		PR	9	0.437
PN17	PR	nd		U-NZpol1	10	0.672
PN18	PR	10	0.000	U-NZpol1	10	0.426
PN21	PR	10#	1.464	U-NZpol1	nd	
PN22	PR	10	1.257	U-NZpol1	nd	
PN23	PR	10#	0.375	U-NZpol1	11	0.658
PN24	C	nd		U-NZpol1	11	1.124
TKP73	U-NZenv	9	0.369	U-NZpol1	12	0.442
TKP88	U-NZenv	11	2.352	U-NZpol1	11	0.447
TKP94	U-NZenv	6	0.691	U-NZpol1	11	5.240
					6 and consensus	1.599
					4	0.661
TKP95	U-NZenv and C	13	25.717**	U-NZpol1	10	2.088
	U-NZenv	10	2.313			
	C	3 and consensus	0.129			

# samples for which endpoint dilution sequencing was documented in Hayward *et al*. (2007), but here the sequence number has been extended from five to ten sequences per sample.

*PR = putative recombinant

**sample with dual infection

^nd = not done

### Intrahost recombination

**Figure 7:**
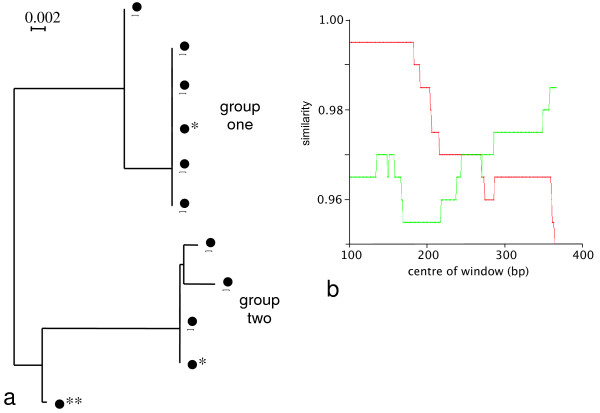
**Intrahost recombination evidence from endpoint dilution sequencing of *pol *gene of TKP94**. (a) NJ tree showing the eleven endpoint dilution sequences for the *pol *gene of TKP94. * represents sequences used as reference sequences in the RIP analysis. ** represents the outlier sequence. (b) RIP output for the outlier sequence. Red is similarity to reference sequence from group one; green is similarity to reference sequence from group two.

One of the exceptions found from endpoint dilution sequencing is the *pol *gene of sample TKP94. Eleven endpoint dilution sequences were obtained for this sample, which fall into two main groups, comprising six and four sequences (Fig. 7a). The eleventh sequence is an outlier between these two groups and thus was submitted to RIP as a check for recombination. The RIP output clearly shows a crossover event between the two main groups (Fig. 7b).

Given the *pol *evolutionary rate of 1–3% per decade, as documented in FIV-Pco, [13], it would take between 17 and 52 years to generate diversity of 5.2% in an individual as seen in the *pol *sequences from sample TKP94 (Table 8). Since this particular individual is only a juvenile, we suggest the explanation for the viral diversity found in this cat is a result of same-subtype dual infection.

**Figure 8:**
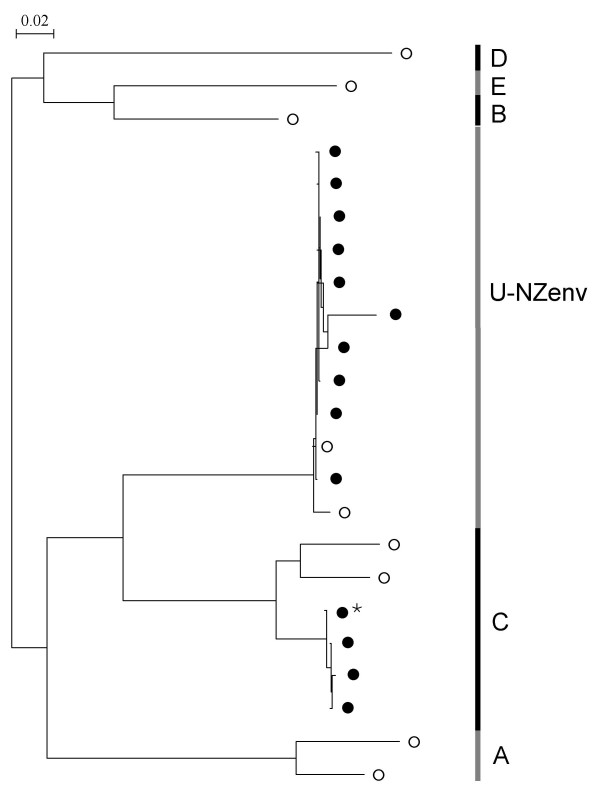
**Dual subtype infection identified from endpoint dilution sequencing of *env *gene of TKP95**. NJ tree showing the thirteen endpoint dilution sequences (●) and the consensus (*) from the *env *gene of TKP95. Reference sequences (○) are included to show subtypes.

The second exception found from endpoint dilution sequencing is seen in the *env *gene of TKP95. These fourteen sequences have a maximum pairwise distance of 25.7%, a value resembling intersubtype distances. Indeed, when a phylogeny is constructed of the TKP95 sequences, we find ten sequences in the U-NZenv group and the remaining three (and the consensus) in subtype C (Fig. 8). This dual infection in the *env *gene of TKP95 is the only case of naturally-occurring dual subtype infection we have found in NZ FIV-infected cats. We have found no evidence of intersubtype recombination in the *env *gene of this dually-infected cat. However, recombinant sequences may be present, but at low frequencies.

### Geographical location

Although approximately equal numbers of companion (n = 72) and feral (n = 77) FIV-infected cats were included in this study, all but one of the twelve putative intragenic recombinants were found in companion cats. Only one, an *env *recombinant, was isolated from a feral cat.

**Figure 9:**
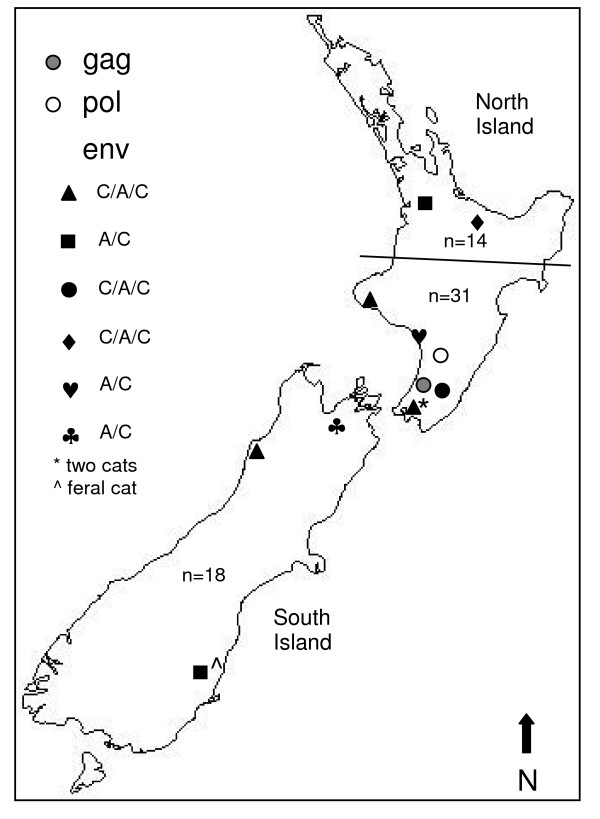
**NZ map showing locations of cats infected with different putative intragenic recombinant sequence crossover patterns**. Sample numbers for cats in the upper half of the North Island, the lower half of the North Island, and the South Island refer to companion cats only.

The locations of the companion cats found to have putative intragenic recombinant FIV sequences are centred around the lower North Island (Fig. 9). However, the lower North Island also has a larger sample size of FIV-infected companion cats. The higher number of recombinants located in the lower North Island with respect to sample size is not significantly different to the upper North Island and the South Island (Fisher Exact Test; P = 0.841).

As we identified only three putative intergenic recombinants in this study, isolated from two feral and one ex-stray cat from three different locations, these were not included in the analysis to determine a geographical pattern.

## Discussion

### Intragenic putative recombination

Of all three genes analysed from NZ FIV-infected cats, we have found that the *env *gene has the highest level of recombination, of 6.4% (n = 156). We suggest this high level could be because the *env *gene encodes the surface glycoproteins, which contain essential recognition sites for the host immune system [22]. Therefore, this region of the viral genome is under relatively high selective pressure.

We have identified ten putative recombinant A/C *env *sequences, based on results of the KH test. Endpoint dilution sequencing on representative putative recombinant *env *sequences confirmed these as real proviral sequences, with no evidence of dual infection. Within the ten sequences, there are six unique crossover patterns, thus there are a minimum of six circulating FIV-Fca *env *recombinant forms in NZ cats.

Within the *env *gene, we have identified several crossover locations that are common to multiple samples. The most common (shared by six recombinant sequences) is located at the end of the V3 region of the gp120 surface glycoprotein. Several synthetic amino acid sequences from within the V3 region have been found to contain neutralisation epitopes [23], one of which includes the most common putative crossover site found in the present study. This may suggest the importance of recombination in viral neutralisation escape.

A second site, shared by two NZ A/C recombinant sequences (214, MF14), and also two Portuguese B/A isolates (150_02LisP and 164_02UZP) [9] that we detected recombination in, is at the start of the V4 region of the surface glycoprotein.

While the different subtypes involved in the recombination patterns from the V4 region suggest this crossover site is a recombination hotspot, the former site from within the V3 region is likely an ancestral recombination event that is circulating in cat populations.

In comparison to *env*, only one putative recombinant sequence (2.1%), from 48 sequences, was found in the *gag *gene, with two crossover events. A previous study on FIV in cats of Canada found four recombinants from 36 sequences (11%) – three A/B recombinants and one A/C recombinant [6]. All four sequences had at least one crossover site in *gag*, but none of these were located at the same site in the sequence as the *gag *crossovers that we found in the NZ *gag *recombinant sequence.

A single (1.1%) putative intragenic B/U-NZpol1 recombinant was also identified in the *pol *gene, although 43 more samples were sequenced in this region than in *gag*. The *pol *recombinant sequence is the only NZ recombinant we have identified with a subtype B section. Since no subtype B has been found in NZ cats to date, we suggest that this recombinant sequence was imported into NZ. This would mean that strains of the U-NZpol1 group are circulating outside NZ. We feel this is plausible, given the finding that Taiwanese *gag *sequences belong to the U-NZgag group.

The lack of *pol *recombinants may be due to the shorter length of the region that we sequenced for this gene, that is, 524 bp compared to 1127 bp in *gag*, and 858 bp in *env*. However, it is also important to note that the *pol *gene encodes important viral enzymes, such as *reverse transcriptase*, thus is relatively conserved [24]. This leads to the expectation of a low recombination rate in the *pol *gene. In addition, it is also difficult to detect recombination in regions of low diversity. No known study has found naturally-occurring intersubtype recombination in the *pol *gene of the FIV-Fca strain.

The twelve NZ intragenic putative recombinants that we have identified were almost exclusively found in companion cats. Due to the design of this study, we are unsure as to why this could be; one hypothesis is that companion cats may represent the reservoir pool of recombinants, which have not yet spread extensively through the feral cat populations. Alternatively, recombinant strains may be more virulent than non-recombinant strains. Infection with a circulating recombinant would result in death in feral cats, while companion cats, with their generally higher standard of living, remain alive but infected. While these two hypotheses are plausible, we feel that neither is fully adequate to explain the situation. A final potential explanation is the sample used for each cat type. Lymph nodes were used for feral cats while blood samples were used for companion cats. This would need to be confirmed by comparing viral sequences in a putative recombinant cat from both lymph node and blood samples.

### Putative intergenic recombination

We found three cases of statistically significant differences between gene trees from the same sample, leading to the conclusion that we have three putative intergenic recombinants. These putative intergenic recombination events are between the *gag *and *env *gene regions sequenced in this study.

The *pol *gene region of only 524 bp did not have enough phylogenetic signal to elevate the power of the SH tests. This is evident in the ML trees, where unresolved nodes are seen. The lack of signal could be a result of the conserved nature of the *pol *gene, given that it encodes important enzymes.

The SH test also is known to be conservative [25]. We likely added to this stringency with the criteria of both reciprocal tests needing to be statistically significant to recognise significance between the two trees involved. However, in this case we feel it is better to be cautious in designating intergenic recombinants.

### Unknown subtype

The *env *unknown subtype samples that we previously found [10] do not show the same pattern in the *gag *and *pol *gene trees, and we cannot confirm the occurrence of a novel NZ-specific subtype across the FIV genome. However, other groups of sequences of unknown subtypes are seen in the *gag *(U-NZgag) and *pol *(U-NZpol1, U-NZpol2) NJ trees. Endpoint dilution sequencing carried out on representatives of *pol *and *env *unknown samples confirms that these are real proviral sequences and not artificial PCR-mediated mosaics. Sequences of U-NZenv, U-NZgag and U-NZpol2 are most closely related to reference subtype C, while sequences of U-NZpol1 are most closely related to reference subtype A. In both the *gag *and the *pol *genes, no NZ sequences group directly with the reference subtype C sequences, although there are only three and two C reference sequences in *gag *and *pol*, respectively. We suggest that U-NZgag and U-NZpol2 represent divergent subtype C viral strains. In the *gag *gene, this group also features Taiwan subtype C sequences, indicating a shared origin for NZ and Taiwan FIV and validating the authenticity of our NZ sequences. Further sequencing of Pacific FIV-Fca isolates in this gene is required to confirm whether the U-NZgag group is a Pacific-wide specific subtype.

We suggest that the patterns seen in the different gene regions of the NZ unknown samples are the result of a number of intergenic recombination events involving known subtype A and undefined unknown subtype strains. HIV-1 "subtype E" has a similar putative mosaic structure and it has been suggested that the *gag *and *pol *genes originate from subtype A while most of the *env *gene and LTR's originate from an undefined subtype E parent [21]. The FIV situation we see here is more complex than the HIV-1 "subtype E" case because, firstly, we have unknown groups in all three gene regions we have sequenced, including two distinct unknown groups (U-NZpol1 and U-NZpol2) in the *pol *gene, and secondly, we have at least four different intergenic recombinant patterns across the three genes we have sequenced. Without full-length proviral sequences, we cannot speculate on the relationship between the unknown groups of the three genes, that is, we are unsure of the number of parental unknown strains represented. Indeed, the identified intergenic recombinant pattern of "unknown" in all three gene regions may actually represent sequencing from the same parent strain.

### Endpoint dilution sequencing

All endpoint dilution sequences from putative recombinant samples grouped within 1.5% of each other and the consensus sequence. However, this does not rule out the possibility of recombination occurring in any of these individuals, although our evidence points toward infection in these cats by circulating recombinant forms. With two exceptions (see next section), all endpoint dilution sequences from unknown subtype samples in the *env *and *pol *genes grouped within 2.4% of each other and the consensus sequence. These results validate the authenticity of the original consensus sequence, and provide no evidence for synthetic PCR-mediated recombination.

### Intrahost recombination

From endpoint dilution sequencing, an intrasubtype *pol *recombinant sequence was identified in an individual, which was also found to have both parent strains. The parent strains are quite distinct, although of the same subtype, indicating dual infection. This type of recombination is difficult to detect, due to the high similarity of the sequences involved. This finding highlights that there is a degree of intrasubtype recombination occurring that largely goes unnoticed. Indeed, to our knowledge, no other study has documented a naturally-occurring dual infection of FIV that has resulted in recombination within the individual.

Another interesting result of endpoint dilution sequencing was the identification of dual infection in the *env *gene of another cat, with subtypes C and unknown. Although dual infection is widely accepted to be a prerequisite of recombination, we found evidence of only this one intersubtype dual infection in our samples. In addition, no recombinant sequences were identified from this dually-infected cat.

A recent study [17] has documented two cases of A/C dual infection in NZ cats, found by using different primers to amplify overlapping sequences of the same samples. This finding was explained by different subtype preferential binding of the primers, with one primer set only amplifying subtype A. We have no reason to suspect preferential subtype binding of the primers used in the present study, given that both subtypes A and C are amplified with these.

However, as a confirmation of subtype designation with our primers, we amplified the V3–V5 region of *env *from a selection of 11 samples with different nested PCR primers, as used by Duarte & Tavares (2006). All eleven sequences grouped with the corresponding original sequence (data not shown). While this finding corroborates our data, it does not completely rule out the possibility of primer preferential binding.

## Conclusion

The level of intergenic and intragenic recombination we have found in NZ FIV-infected cats highlights the importance of recombination in the evolution of FIV-Fca. Although this phenomenon has been suggested to occur at a much slower rate than in HIV-1 [26], our results indicate that it may in fact be a more common occurrence, especially given that a lot of recombination goes undetected.

In NZ, we have evidence of FIV-Fca sequences of unknown subtypes in all three *gag*, *pol *and *env *genes. However, the present study represents the most extensive sequencing effort of FIV-Fca strains. Therefore, as expected, and as has been found with increased sequencing of HIV-1 isolates [27,28], we find an increase in the number of strains of unknown subtype. In order to obtain a more accurate picture of recombination and unknown subtype patterns, it is necessary to sequence multiple genes and ideally, to obtain sequences of the whole FIV genome.

Recombination can repair reverse transcription errors [29], or can modify viral properties, such as fitness, as found in HIV-1 BF recombinants circulating in Argentina [30]. The event of recombination can also aid in adaptation to new environments, such as a new host species [31]. Finally, viral recombination increases the genetic diversity of circulating viruses within a population. Results of the present study have implications for the use of the current FIV vaccine. Further testing of, and possibly modifications to, the current vaccine may be needed to ensure protection against circulating recombinant forms and novel subtypes.

## Methods

### Sample collection

Lymph nodes from feral and stray cats, and blood samples from companion cats were collected and processed as described previously [10]. Further samples were collected to give a total of 77 feral cat and 72 companion cat FIV-infected samples included in this study. No further stray cat samples were collected, leaving only the seven stray cat sequences from our previous study. For this study, feral cats are defined as unowned cats that inhabit rural areas, while stray cats inhabit urban areas. Companion cats are owned and fully reliant on humans.

### PCR amplification

The *env *gene was amplified using nested PCR, according to published protocols [10,32]. In addition to the *env *gene, a region of the *gag *gene [6] and a region of the *pol *gene [33] were also directly sequenced for some samples (Table 9). Forty-eight (32%) of the samples were amplified successfully with the *gag *primers and 91 (61%) were amplified successfully with the *pol *primers. Sequencing and sequence analyses were conducted as previously described [10]. NZ sequences generated in this study were deposited in GENBANK.

**Table 9:** *Env*, *gag *and *pol *primer details used in the nested PCR's

Gene region and PCR round	Primer Name	Location (bp)	PCR product length (bp)
*env *– round one	VE1S	7134–7154*	1230
	VE1R	8364–8345	
- round two	VE2S	7326–7345	858
	VE2R	8184–8165	
*gag *– round one	LTR1	122–141**	1287
	Gag548	1409–1389	
- round two	LTR2	285–309	1127
	Gag548	1409–1389	
*pol *– round one	1258F	2430–2451**	577
	1260R	3007–2988	
- round two	1259F	2466–2488	524
	1261R	2990–2968	

* using TM2 as reference sequence

** using Petaluma as reference sequence

### Phylogenetic analysis

Using PAUP* [34], a Neighbour-Joining (NJ) tree [35] was constructed for each gene, with the best model of evolution for the data as determined by Modeltest 3.7 [36]. For *env*, *gag *and *pol *the optimum model was GTR+I+G.

### Intragenic recombination identification

The NJ trees were used to visually identify sequences that fall between subtypes (outliers), which were then tested for recombination using Recombinant Identification Program 3.0 (RIP) [37,38]. A 200 bp sliding window was used, with the threshold for significance set at 90%. Gaps in the sequences were treated as characters and multistate characters were scored as partial matches. Appropriate reference sequences were included in each RIP analysis.

Each outlier sequence was then split at the putative crossover points deduced from the RIP alignment output. In PAUP* a maximum likelihood (ML) tree was constructed using each sequence "half" and full-length reference sequences, with a backbone tree of full-length reference sequences constrained. The KH test [39,40], also implemented in PAUP*, was used to test for a significant difference between the two ML trees built from each sequence "half" of each recombinant.

### Intergenic recombination identification

Forty-five NZ cat samples were sequenced in all three gene regions. Locations of each of these 45 samples on the different gene trees were compared. Six samples are identified as intragenic putative recombinant sequences in this study and were removed from the data. This was done to prevent a significant result caused by the intragenic recombination event. Nine of our remaining samples showed a discrepancy in subtype assignment between gene trees, indicating intergenic recombination.

If there is no recombination, we expect that a single topology applies to all gene regions. The best candidate for this topology is obtained by concatenating all gene alignments, and constructing a tree. We did this as follows. All intergenic putative recombinant sequences were removed from the data, leaving a total of 30 NZ sequences, from which four ML trees (*gag *gene, *pol *gene, *env *gene and concatenation of all three genes) were constructed. Modeltest output parameters were used (GTR+I+G for *gag *and concatenated, TrN+G for *pol*, TVM+I+G for *env*) in a heuristic search with random sequence addition, ten replicates and tree bisection reconnection as the branch-swapping algorithm. We used the Shimodaira-Hasegawa (SH) test [41] to determine whether the concatenated tree is an appropriate backbone constraint to control for sampling error in the topologies of the three different gene regions. The SH test was implemented in PAUP*, with full optimisation and 1000 bootstrap replicates. The null hypothesis in an SH test is that all the trees being compared are just as good at explaining the data as the true tree. A statistically significant result rejects the null hypothesis, indicating that the particular tree does not explain the data as well as the best tree for that data set [25]. Four SH tests were run – one for each data set (*gag*, *pol*, *env *and concatenated). Therefore, a Bonferroni correction was applied.

SH test results showed that the concatenated tree is not significantly different to the best tree for each gene data set. A putative recombinant sequence was then added into the data and four ML trees (*gag *gene, *pol *gene, *env *gene and concatenation of all three genes) were estimated, with the original concatenated tree constrained as a backbone. A heuristic search was implemented, with random sequence addition, ten replicates and tree bisection reconnection as the branch-swapping algorithm. Four ML trees were constructed for each of the nine identified putative intergenic recombinant sequences. Four data sets (168 *pol*, WST05 concatenated, WST05 *gag*, 259 *pol*) returned multiple trees, but as these trees all had the same likelihood score, one of the trees was randomly selected for use in subsequent SH tests.

Since the concatenated tree is constrained as a backbone, the only differences between the four ML trees are the locations of the newly added putative intergenic recombinant sequence and the branch lengths. So, any statistical significance found from the SH tests is a result of the location of the putative recombinant sequence. In this way, we are testing the putative recombinant sequences for incongruence between the different gene regions. The SH test was performed using PAUP* with full optimisation and 1000 bootstrap replicates, as described in Goldman *et al. *(2000). For each of the putative recombinant sequences, the SH test was run four times – once with each data set (*gag*, *pol*, *env*, concatenated). A Bonferroni correction was applied to correct for the multiple tests. A sequence was designated an intergenic recombinant if the corresponding trees were significantly different statistically in both reciprocal data set SH tests. With nine putative recombinants and four trees for each, a total of 36 SH tests were run. Note that SH tests were run to compare all gene regions, not just those we noticed an incongruency between.

### Endpoint dilution

Endpoint dilution sequencing [42,43] was undertaken as described in Hayward *et al*. (2007) on representative intragenic putative recombinants and unknown subtype samples, for both the *env *and *pol *genes. Endpoint dilution was also carried out on the putative recombinant sample of the *gag *gene. This was done to confirm the consensus sequences generated from direct sequencing as actual proviral sequences and not PCR-mediated recombinants as a result of dual infection [44,45].

## Competing interests

The authors declare that they have no competing interests.

## Authors' contributions

JJH was involved in the study design, conducted the lab work, performed the analyses and drafted the manuscript, AGR was involved in conception and design of the study, design of analyses and revision of the manuscript.

Both authors read and approved the final manuscript.
